# Regional vulnerability of brain white matter in vanishing white matter

**DOI:** 10.1186/s40478-023-01599-6

**Published:** 2023-06-22

**Authors:** Jodie H.K. Man, Charlotte A.G.H. van Gelder, Marjolein Breur, Douwe Molenaar, Truus Abbink, Maarten Altelaar, Marianna Bugiani, Marjo S. van der Knaap

**Affiliations:** 1 Department of Child Neurology, Emma Children’s Hospital, Amsterdam University Medical Centers, VU University Amsterdam, Amsterdam, 1081 HV The Netherlands; 2 Amsterdam Leukodystrophy Center, Emma Children’s Hospital, Amsterdam University Medical Centers, Amsterdam, 1081 HV The Netherlands; 3 Molecular and Cellular Mechanisms, Amsterdam Neuroscience, Amsterdam, 1081 HV The Netherlands; 4 Biomolecular Mass Spectrometry and Proteomics, Bijvoet Center for Biomolecular Research and Utrecht Institute for Pharmaceutical Sciences, University of Utrecht, Utrecht, 3584 CS The Netherlands; 5 Netherlands Proteomics Center, Utrecht, 3584 CS The Netherlands; 6 Department of Systems Bioinformatics, VU University Amsterdam, Amsterdam, 1081 HV The Netherlands; 7 Department of Pathology, Amsterdam University Medical Centers, Amsterdam, 1081 HV The Netherlands; 8 Department of Integrative Neurophysiology, Center for Neurogenomics and Cognitive Research, VU University Amsterdam, Amsterdam, 1081 HV The Netherlands

**Keywords:** Vanishing white matter, Leukodystrophy, Astrocytopathy, Regional vulnerability, Proteomics

## Abstract

Vanishing white matter (VWM) is a leukodystrophy that primarily manifests in young children. In this disease, the brain white matter is differentially affected in a predictable pattern with telencephalic brain areas being most severely affected, while others remain allegedly completely spared. Using high-resolution mass spectrometry-based proteomics, we investigated the proteome patterns of the white matter in the severely affected frontal lobe and normal appearing pons in VWM and control cases to identify molecular bases underlying regional vulnerability. By comparing VWM patients to controls, we identified disease-specific proteome patterns. We showed substantial changes in both the VWM frontal and pons white matter at the protein level. Side-by-side comparison of brain region-specific proteome patterns further revealed regional differences. We found that different cell types were affected in the VWM frontal white matter than in the pons. Gene ontology and pathway analyses identified involvement of region specific biological processes, of which pathways involved in cellular respiratory metabolism were overarching features. In the VWM frontal white matter, proteins involved in glycolysis/gluconeogenesis and metabolism of various amino acids were decreased compared to controls. By contrast, in the VWM pons white matter, we found a decrease in proteins involved in oxidative phosphorylation. Taken together, our data show that brain regions are affected in parallel in VWM, but to different degrees. We found region-specific involvement of different cell types and discovered that cellular respiratory metabolism is likely to be differentially affected across white matter regions in VWM. These region-specific changes help explain regional vulnerability to pathology in VWM.

**Supplementary Information:**

The online version contains supplementary material available at 10.1186/s40478-023-01599-6.

## Introduction

Vanishing white matter (VWM, OMIM #603,896) is one of the more prevalent leukodystrophies. It may present at any age, but primarily manifests in children [39]. VWM is caused by autosomal recessive pathogenic variants in any of the 5 genes encoding the subunits of the eukaryotic translation initiation factor 2B (eIF2B) [22, 40]. This is a guanine nucleotide exchange factor for eIF2 and serves as a critical component for the initiation of translation. eIF2B activity is tightly regulated via the activation of the integrated stress response (ISR), a control pathway that protects cells in response to cellular stress [29, 43]. In VWM, there is constitutive deregulation of the ISR [1]. Clinically, the disease is characterized by chronic neurological decline with patients being susceptible to stressors that trigger episodes of rapid deterioration. These episodes may be followed by partial recovery or early death [6, 37, 41].

Typical patterns of VWM are dominated by degeneration of white matter structures with meagre reactive gliosis, lack of myelin, axonal abnormalities, and immature astrocytes and oligodendrocytes [5–7, 9, 11, 20, 37, 41]. Although eIF2B is a ubiquitous factor, a striking feature of VWM is that the disease is more prominent in certain brain white matter regions than in others [9]. Imaging and neuropathology studies show that the cerebral white matter is rarefied and cystic. The cerebellar white matter is also affected, but with little to no tissue loss. Involvement of the white matter in the brainstem may also occur, but is often minimal [9, 37]. Thus, studies show a remarkable and consistent regional variability in disease severity with telencephalic areas such as the frontal lobe most severely affected and relentlessly degenerated, whereas other areas like the brainstem are remarkably, often completely spared. Our understanding of the molecular bases contributing to this regional vulnerability is still poor.

It is important to elucidate how these differential regional vulnerabilities arise in order to understand VWM pathology. Here we aimed at gaining more insight into the molecular bases underlying selective regional vulnerability of the brain white matter in VWM. We performed a high-resolution mass spectrometry-based proteome analysis of 2 differently affected brain regions in VWM, that is, the white matter of the frontal lobe and the pons. Regional protein expression patterns were analyzed and compared to identify disease mechanisms that may contribute to regional vulnerability in VWM.

## Materials and methods

### Patients

This study focused on the white matter of the middle frontal gyrus and the mid-pons. Post mortem brain tissue from 4 genetically proven VWM patients was collected at the Amsterdam University Medical Centers location VU University Amsterdam (Amsterdam, The Netherlands). Patients were selected based on the severity of the white matter pathology (i.e., white matter not completely vanished, also in the frontal lobe). In addition, 4 controls obtained from the Netherlands Brain Bank were included. No confounding neuropathological or structural abnormalities were found in these controls. Tissue was obtained within 6 h post mortem. Informed consent was obtained in all cases. The study was approved by the Medical Ethical Committee of the Amsterdam University Medical Centers location VU University Amsterdam (Amsterdam, The Netherlands) and conducted according to the declaration of Helsinki. Demographic features of controls and patients are shown in Table 1.

**Table 1:** Demographic features of controls and patients

Cases	Age at death (years)	Molecular diagnosis	Brain areas analyzed
Control 1	24	None	Frontal
Control 2	23	None	Frontal, pons
Control 3	21	None	Pons
Control 4	35	None	Frontal, pons
Patient 1	29	ε, Thr91Ala/Thr91Ala*	Frontal, pons
Patient 2	10	ε, Arg113His/Ala403Val*	Frontal, pons
Patient 3	6	ε, Thr91Ala/Val437Met*	Frontal, pons
Patient 4	12	ε, Thr91Ala/Ala403Val*	Frontal, pons

*Mutation: mutant *eIF2B* subunit and amino acid changes are indicated.

### Tissue preparation and laser microdissection

Fresh frozen tissue sections of 20µm thickness were mounted on polyethylene naphthalate-coated glass slides, fixed in 100% ethanol for 20 min, air-dried and rehydrated twice in sterile H_2_O for 1 min. Next, tissue sections were stained using toluidine blue (1% *w/v* in sterile H_2_O) for 1 min at room temperature and washed 3 times in sterile H_2_O for 1 min. Afterwards, sections were dehydrated in 100% ethanol twice for 3 min and air-dried. Brain regions of interest were then microdissected using a Leica LMD6500 system (Leica Microsystems). A volume of 100mm^3^ of white matter tissue from the frontal lobe and the pons was collected into adhesive caps (Zeiss). Microdissected tissues were stored at -80℃ until further use.

### Sample preparation for mass spectrometry analysis

Microdissected tissue samples were incubated in lysis buffer (6 M guanidine hydrochloride, 5 mM tris(2-carboxyethyl)phosphine, 10 mM chloroacetamide and 100 mM Tris-HCl in 50 mM ammonium bicarbonate) for 10 min at 99 °C with mixing at 750RPM. Tissue lysates were then sonicated with 20 mg protein extraction beads (Diagenode) using a Bioruptor Plus (Diagenode) for 20 cycles with on/off pulses of 30 s. Protein digestion was performed using Lys-C in a 1:100 enzyme-to-substrate ratio at 37 °C for 4 h. Afterwards, samples were diluted to a final concentration of 2 M guanidine hydrochloride followed by overnight digestion with trypsin at a 1:100 enzyme-to-substrate ratio at 37 °C. Digestion was quenched by lowering the pH below 2 using 10% formic acid. Peptide samples were then desalted using C18 cartridges (Agilent Technologies) on the automated AssayMAP Bravo Platform (Agilent Technologies). Cartridges were first primed and equilibrated using 80% acetonitrile/0.1% formic acid and 0.1% formic acid, respectively. Peptides were then loaded and washed with 0.1% formic acid, and eluted with 80% acetonitrile/0.1% formic acid. Eluted peptides were dried using a SpeedVac centrifuge and stored at − 20 °C.

### Mass spectrometry analysis and processing

Mass spectrometry analysis was conducted on an Orbitrap Q-Exactive HF-X mass spectrometer (Thermo Fisher Scientific) coupled to an UltiMate 3000 RSLCnano System (Thermo Fisher Scientific). Peptides (1.5 µg/sample) were resuspended in 2% formic acid and loaded on a trap column (300 μm i.d. x 5 mm, 5 μm particle size, reversed phase C18, Thermo Fisher Scientific) at a flow rate of 10 µL/min in 100% buffer A (0.1% formic acid). Peptides were separated on a self-packed analytical column. Elution was performed over a 175 min gradient at a flow rate off 300 nL/min with buffer B (80% acetonitrile/0.1% formic acid): 0–2.5 min 92% solvent A; 2.5– 57.5 min 8% solvent B; 157.5–161 min 35% solvent B; 161–165 min 100% solvent B; 165–175 min 92% solvent A. Data-dependent acquisition method was used to collect mass spectra. Briefly, first a full MS1 scan (m/z range = 315–1,500, resolution = 60,000, target = 3 × 10^6^ ions) was acquired. Then the top 15 precursors were selected for MS2 analysis with higher-collision dissociation (target ions = 1 × 10^5^, max ion fill time = 50 milliseconds, isolation window = 1.4 m/z, normalized collision energy = 27%, resolution = 30,000). Dynamic exclusion was set to 16 s. Precursor ions with unassigned charge state as well as charge state of 1 and 6 or higher were excluded from fragmentation.

### Label-free quantification

RAW mass spectra files were searched against the Swiss-Prot human reference proteome database (20,431 entries, accessed on August 2019) and analyzed using MaxQuant (v1.6.6.0) [36]. “Match between runs” was enabled and the false discovery rate (FDR) threshold was set to 1%. Label-free quantification (LFQ) was conducted using the MaxLFQ algorithm with a minimal ratio count of 2. Carbamidomethylation of cysteine was set as fixed modification. For variable modifications, peptide N-terminal acetylation and methionine oxidation were selected. Furthermore, only peptides with a maximum of 2 trypsin cleavages, length ranging from 7 to 25 amino acids, mass tolerance of 20ppm, and fragmentation mass tolerance of 0.5Da were included for analysis.

### Differential protein expression analysis

Statistical analysis was performed using the R statistical software (v1.3.1093). Differential protein expression analysis was analyzed using DEP R package [44]. Peptides recognized as contaminants, only by site modification or as decoy in the reverse database were excluded from the analysis. LFQ intensity values were then log_2_-transformed. Only proteins detected in all replicates of at least one condition were considered for further analysis. Missing data were imputed using random draws from a left-shifted Gaussian distribution centered around a minimal mean value being the lowest observed expression value. The standard deviation was estimated as being the mean standard deviation of all detected proteins. Statistical significance was set to *q*-value < 0.05 with Benjamini-Hochberg’s (BH) adjustment for multiple comparison. Principal component analysis was conducted using FactoMineR R package [21]. A Pearson’s correlation analysis of proteins with significant differential expression across regions analyzed was performed to determine regional similarities or differences.

### Expression-weighted cell type enrichment analysis

Cell type enrichment analysis was performed using the EWCE R package based on a single nuclei RNA sequencing dataset of 5291 cells from brain white matter tissue of 3 controls [2, 33]. These cells were divided into 7 clusters including astrocytes, immune cells (i.e., microglia and macrophages), lymphocytes, neurons, oligodendrocytes, oligodendrocyte progenitor cells (OPCs), and vascular cells [2]. All proteins detected in our proteome analysis were used as the background set. Analysis was performed with 10,000 bootstrap lists. Statistical significance was set to *q*-value < 0.05 with BH adjustment for multiple comparison.

### Overrepresentation analysis

Functional gene ontology overrepresentation analysis was conducted using PANTHER Classification System (version 17.0) with a Fisher’s exact test [25, 35]. The following gene ontology databases were included: biological process, cellular component, and molecular function. For visualization, only the most specific subclasses are shown, which were selected by default hierarchical sorting. For biological pathway analysis, we used the Kyoto Encyclopedia of Genes and Genomes (KEGG) database in combination with g:Profiler (version 107_eg54_p17_bf4221) [17–19, 31]. Statistical significance for both analyses was set as *q*-value < 0.05 with BH adjustment for multiple comparison. All proteins detected in our proteome analysis were set as background.

## Results

To gain insight into selective regional vulnerability in VWM, we conducted a high-resolution mass spectrometry-based proteome analysis on the white matter of the frontal lobe and the pons, the first representing a severely affected region and the latter being relatively spared. Four controls and 4 VWM patients with childhood-onset were included in the study (Table 1). Patients were selected based on severity of the affected white matter of the frontal lobe, and normal imaging and neuropathology of the pons as described by earlier studies (Supplemental Fig. 1) [9, 37].

**Fig. 1:**
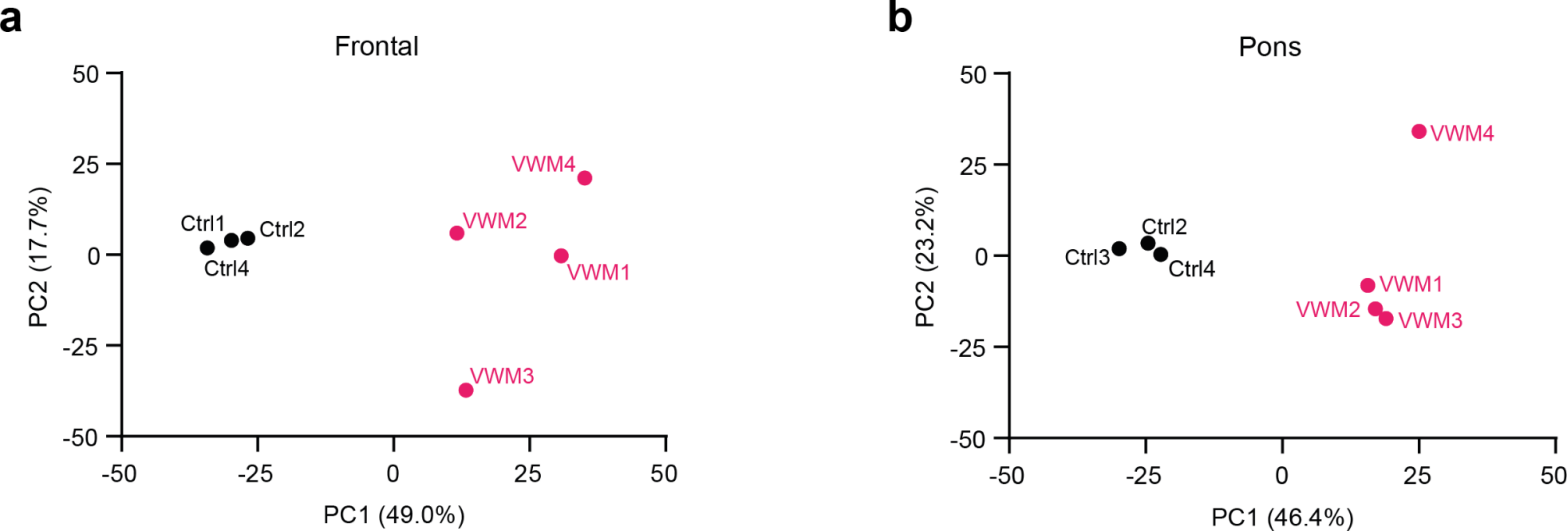
Principal component analyses of the white matter in the frontal lobe and pons in control and VWM cases. (**a**) Analysis of the frontal white matter shows that the first component separates control cases (black) from VWM cases (pink) and accounts for 49.0% of the variability. The second component reveals segregation between VWM cases and explains 17.7% of the variability. (**b**) Analysis of the pons white matter reveals separation of VWM patients (pink) and controls (black) in the first component, explaining 46.4% of the variability. Only one patient sample deviates from the patient group as revealed by the second component. This accounts for 23.2% of the variability. All controls aggregated together across regions. *PC1* principal component 1, *PC2* principal component 2, *Ctrl* control, *VWM* vanishing white matter

Proteome analysis identified an average of 22,053 peptides per sample. Missing values were imputed, mapping to a total of 3149 proteins across 14 samples (Supplemental Table 1). Principal component analyses showed clear separation of controls from VWM cases in the white matter of both the frontal lobe and the pons. Control cases in both regions formed distinct clusters, where samples from biological replicates strongly aggregated. Remarkably, in the frontal white matter, patient samples segregated from each other, indicating some degree of between-patient variation. In the pons, only one patient sample deviated from other biological replicates (Fig. 1). Principal component analysis of all samples together showed that control samples obtained from different brain regions were separated from each other, reflecting region-specific differences. Additionally, VWM cases region-dependently formed distinct clusters from controls. VWM frontal white matter samples were strongly separated from other samples. Notably, VWM pons white matter samples closely clustered with frontal white matter samples from control cases (Supplemental Fig. 2).

**Fig. 2:**
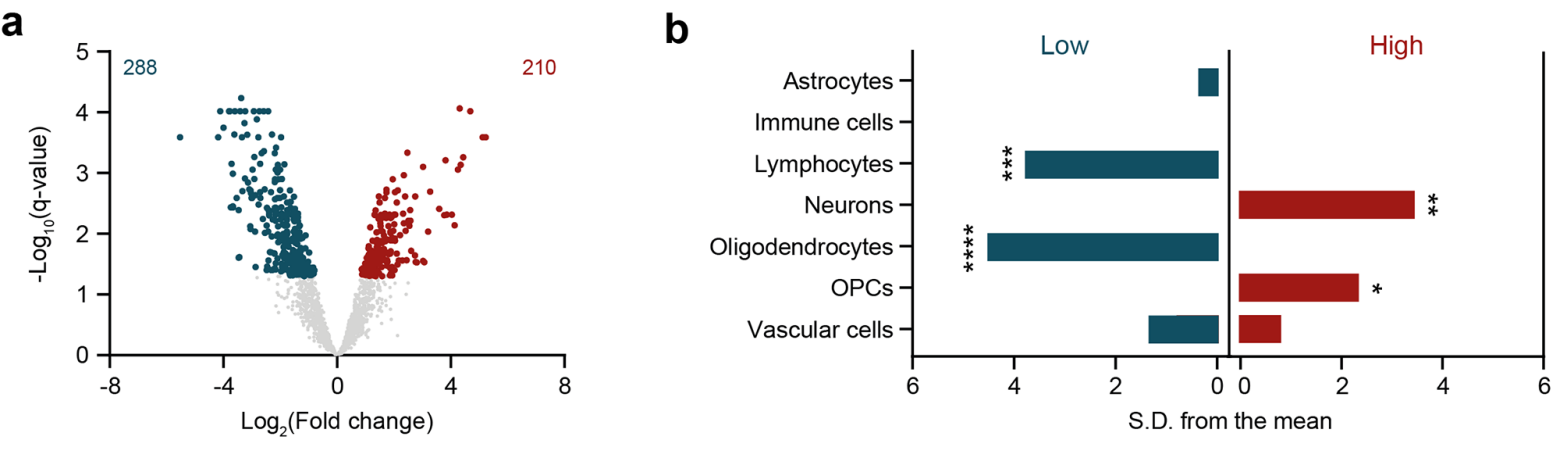
Proteome pattern of the frontal white matter in VWM. (**a**) Differential expression analysis reveals proteome differences in the frontal white matter of controls compared to VWM patients. Volcano plot shows proteins with significantly lower (blue, left) and higher (red, right) expression in the VWM frontal white matter (*q*-value < 0.05). Not significantly differentially expressed proteins (*q*-value > 0.05) are colored in gray. (**b**) EWCE analysis indicate that lower (blue, left) and higher (red, right) expressed proteins in the frontal white matter of VWM patients are associated with distinct cell types. * *q*-value < 0.05, ** *q*-value < 0.01, *** *q*-value < 0.001, **** *q*-value < 0.0001, *S.D.* standard deviation, *OPCs* oligodendrocyte progenitor cells

### Proteome pattern of the frontal white matter in VWM

Proteome analysis of the white matter of the frontal lobe revealed a total of 498 significantly differentially expressed proteins (*q*-value < 0.05). Amongst these, 288 were downregulated and 210 upregulated (Fig. 2a and Supplemental Table 2). The top 10 significantly differentially expressed proteins ranked by fold change are listed in Table 2. Among the deregulated proteins, we observed several previously associated with VWM white matter pathology [9]. For example, we found reduction in expression of myelin basic protein (MBP, *p =* 0.0037) and myelin proteolipid protein (PLP1, *p* = 0.0200), as well as upregulation of the intermediate filament nestin (NES, *p* = 0.0049) and vimentin (VIM, *p* = 0.0653) (Supplemental Table 2). We next examined whether these changes in protein expression were related to specific cell types using an expression-weighted cell type enrichment (EWCE) analysis. Amongst the proteins with decreased expression in the frontal white matter of VWM patients, we found a significant enrichment for lymphocytes (*q*-value = 0.00035) and oligodendrocytes (*q*-value < 0.0001), whereas proteins with increased expression were enriched for neurons (*q*-value = 0.0042) and OPCs (*q*-value = 0.04) (Fig. 2b).

**Table 2:** Top 10 significantly differentially expressed proteins in the white matter of the frontal lobe in VWM

Direction	Gene symbol	Protein name	Log_2_ (Fold change)	*q*-value
Upregulated	BSN	Protein bassoon	5.233	0.000258
Upregulated	ATP6V0A1	V-type proton ATPase 116 kDa subunit a isoform 1	5.123	0.000258
Upregulated	L1CAM	Neural cell adhesion molecule L1	4.688	0.000096
Upregulated	SV2A	Synaptic vesicle glycoprotein 2 A	4.443	0.000550
Upregulated	SYNM	Synemin	4.354	0.000733
Upregulated	NPTN	Neuroplastin	4.311	0.000086
Upregulated	ATP2B1	Plasma membrane calcium-transporting ATPase 1	4.254	0.000875
Upregulated	PSD3	PH and SEC7 domain-containing protein 3	4.137	0.007253
Upregulated	RAB3D	Ras-related protein Rab-3D	4.039	0.004826
Upregulated	COL6A3	Collagen alpha-3(VI) chain	3.861	0.004865
Downregulated	LGALS1	Galectin-1	-3.664	0.001022
Downregulated	GLUL	Glutamine synthetase	-3.666	0.003525
Downregulated	ALDH4A1	Delta-1-pyrroline-5-carboxylate dehydrogenase, mitochondrial	-3.709	0.000707
Downregulated	OXR1	Oxidation resistance protein 1	-3.736	0.003678
Downregulated	VCAN	Versican core protein	-3.771	0.000096
Downregulated	PRDX6	Peroxiredoxin-6	-3.798	0.000096
Downregulated	BASP1	Brain acid soluble protein 1	-3.996	0.000179
Downregulated	ERMN	Ermin	-4.115	0.000096
Downregulated	GLTP	Glycolipid transfer protein	-4.185	0.000258
Downregulated	ALDH7A1	Alpha-aminoadipic semialdehyde dehydrogenase	-5.528	0.000258

**Fig. 3:**
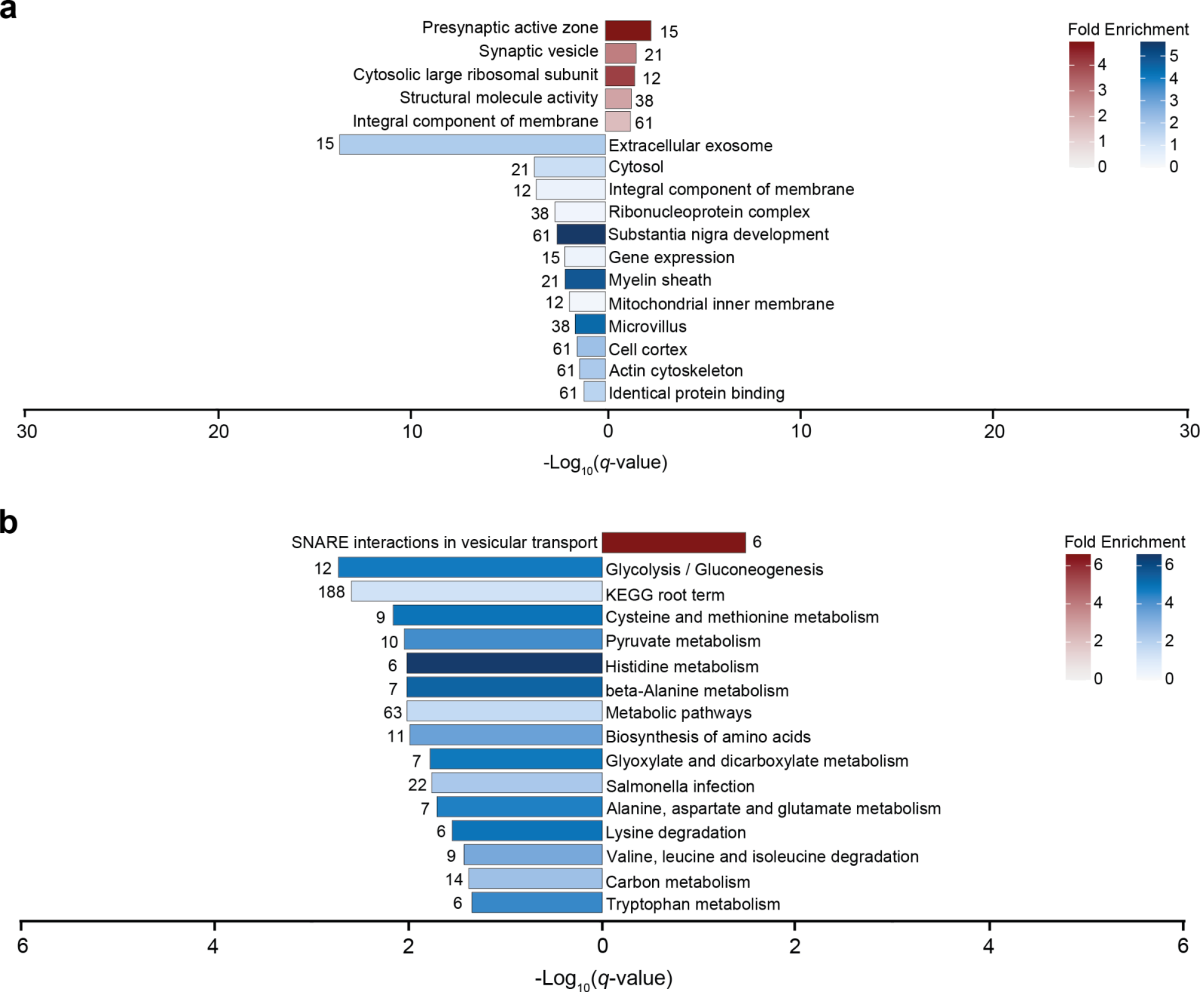
Functional and pathway analysis of deregulated proteins in the frontal white matter of VWM patients. (**a**) Gene ontology and (**b**) KEGG pathway analysis of downregulated (n = 288) and upregulated (n = 210) proteins. In (**a**), only the most specific ontology subclasses are shown. In (**a-b**), red and blue bars represent terms associated with higher and lower expressed proteins, respectively. Number of proteins annotated in each term is listed in the plot. Fold enrichment values below 1 represent underrepresented terms, whereas a value above 1 represents overrepresented terms

We then sought to determine which biological functions are modulated by these protein changes by means of functional gene ontology overrepresentation analysis. Proteins with increased or decreased expression were separately analyzed. For the proteins with increased expression, we found enrichment for terms related to synaptic transmission (Fig. 3a). Proteins with lower expression were enriched for terms related to brain development, myelin sheath, cell structure, and extracellular exosome (Fig. 3a). Interestingly, this analysis also revealed terms that were underrepresented. These were associated with gene expression, ribonucleoprotein complex, and membrane (Fig. 3a).

Pathway analysis of the significantly deregulated proteins using the KEGG database revealed that some proteins with enhanced expression were enriched for SNARE interactions in vesicular transport, suggestive of alterations in vesicle transport (Fig. 3b). Proteins with decreased expression were enriched for glycolysis/gluconeogenesis, amino acid metabolism, and glyoxylate and dicarboxylate metabolism, reflecting changes in cellular metabolism (Fig. 3b). Results of the functional overrepresentation and pathway analyses are provided in Supplemental Table 3.

### Proteome pattern of the pons white matter in VWM

**Fig. 4:**
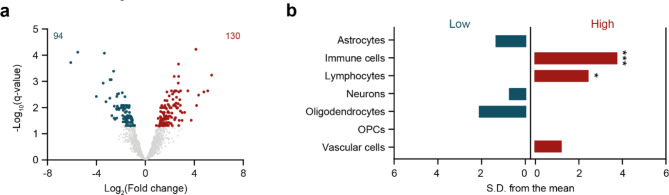
Proteome pattern of the pons white matter in VWM. (**a**) Differential expression analysis reveals proteome differences in the pons white matter of controls compared to VWM patients. Volcano plot shows proteins with significantly lower (blue, left) or higher (red, right) expression in VWM pons white matter (*q*-value < 0.05). Not significantly differentially expressed proteins (*q*-value > 0.05) are colored in gray. (**b**) EWCE analysis suggests that upregulated proteins in the pons white matter of VWM patients are associated with distinct cell types. * *q*-value < 0.05, *** *q*-value < 0.001, *S.D.* standard deviation, *OPCs* oligodendrocyte progenitor cells

Proteome analysis revealed a total of 224 significant protein changes in the white matter of the pons in VWM (*q*-value < 0.05), of which 94 were downregulated and 130 upregulated (Fig. 4a and Supplemental Table 2). Table 3 lists the top 10 significantly differentially expressed proteins ranked by fold change. Notably, amongst the proteins known to be affected in VWM [9], we confirmed a significant increase and decrease in NES (*p* = 0.0024) and MBP (p = 0.0095) expression, respectively. Proteins with higher expression were significantly enriched for immune cells (*q*-value = 0.0007), that is microglia and macrophages, and lymphocytes (*q*-value = 0.0252), whereas proteins with low expression were not significantly enriched for a particular cell type (Fig. 4b).

**Table 3:** Top 10 significantly differentially expressed proteins in the white matter of the pons in VWM

Direction	Gene symbol	Protein name	Log_2_ (Fold change)	*q*-value
Upregulated	ANXA1	Annexin A1	5.475	0.000533
Upregulated	FLNC	Filamin-C	5.153	0.002169
Upregulated	NES	Nestin	4.832	0.002385
Upregulated	SCIN	Adseverin	4.397	0.003139
Upregulated	C12orf10	UPF0160 protein MYG1, mitochondrial	4.229	0.007986
Upregulated	PREP	Prolyl endopeptidase	4.177	0.000053
Upregulated	SERPINA1	Alpha-1-antitrypsin;Short peptide from AAT	3.782	0.029830
Upregulated	IQGAP1	Ras GTPase-activating-like protein IQGAP1	3.509	0.002169
Upregulated	FGA	Fibrinogen alpha chain;Fibrinopeptide A;Fibrinogen alpha chain	3.242	0.014698
Upregulated	COL6A3	Collagen alpha-3(VI) chain	3.031	0.030125
Downregulated	ATP6V1G2	V-type proton ATPase subunit G 2	-2.784	0.019741
Downregulated	NCAN	Neurocan core protein	-2.842	0.000796
Downregulated	VCAN	Versican core protein	-2.935	0.000796
Downregulated	SVIP	Small VCP/p97-interacting protein	-2.958	0.004164
Downregulated	ATPIF1	ATPase inhibitor, mitochondrial	-3.278	0.005709
Downregulated	COX7C	Cytochrome c oxidase subunit 7 C, mitochondrial	-3.412	0.000076
Downregulated	AQP1	Aquaporin-1	-3.502	0.001082
Downregulated	PMP2	Myelin P2 protein	-4.073	0.003569
Downregulated	NIN	Ninein	-5.611	0.000069
Downregulated	SYP	Synaptophysin	-6.191	0.000174

**Fig. 5:**
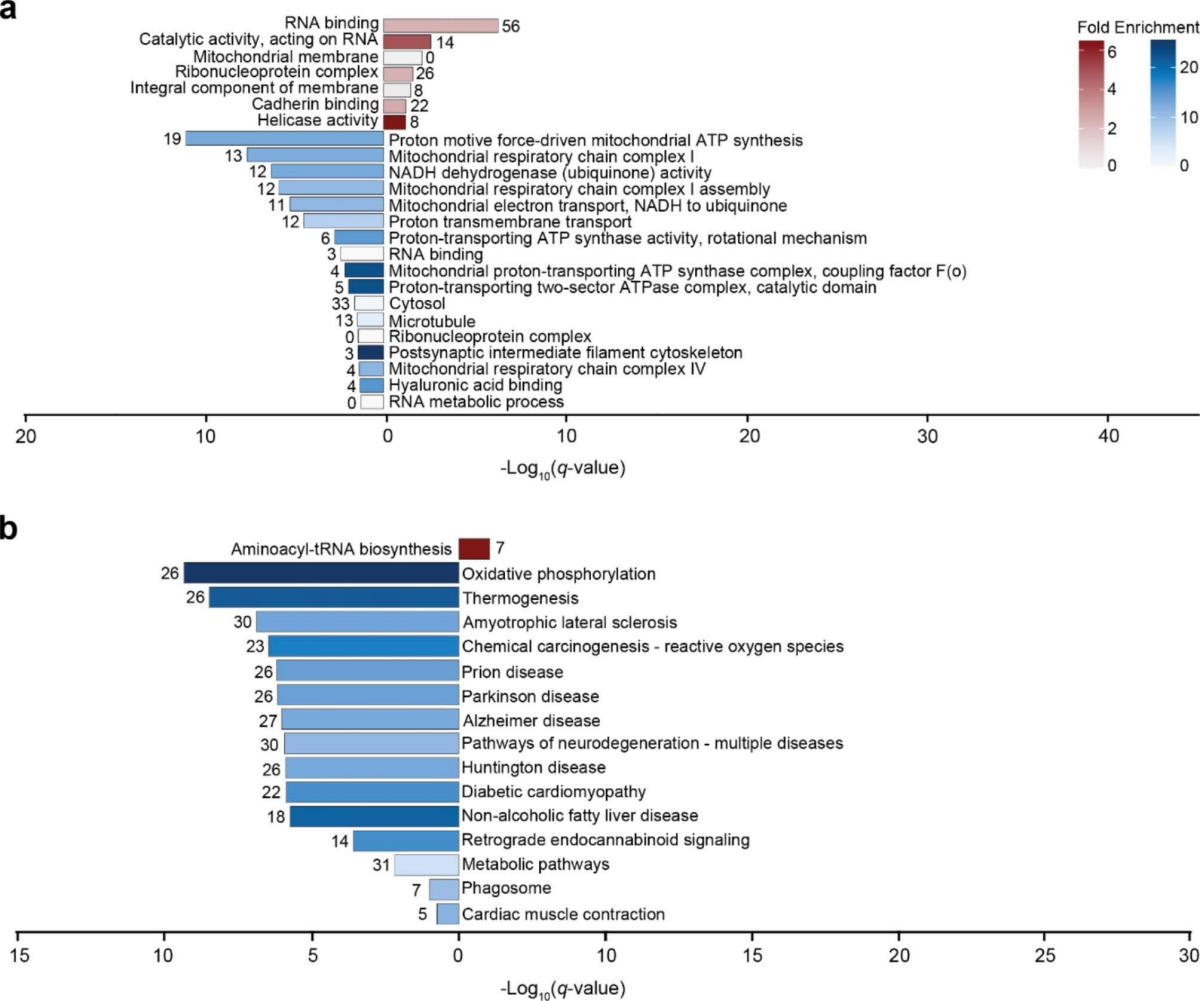
Functional and pathway analysis of deregulated proteins in the pons white matter of VWM patients. (**a**) Gene ontology and (**b**) KEGG pathway analysis of downregulated (n = 94) and upregulated (n = 130) proteins. In (**a**), only the most specific ontology subclasses are shown. In (a-b), red and blue bars represent terms associated with higher and lower expressed proteins, respectively. Number of proteins annotated in each term is listed in the plot. Fold enrichment values below 1 represent underrepresented terms, whereas a value above 1 represents overrepresented terms

Gene ontology overrepresentation analysis of proteins with enhanced expression revealed that terms related to nucleic acid-linked processes were overrepresented. Terms associated with organelle membranes were underrepresented (Fig. 5a). Of the proteins with decreased expression in the pons white matter in VWM, a majority of the overrepresented terms were associated with mitochondrial functioning. Amongst these were terms associated with electron transport and respiratory chain complex. Notably, underrepresented terms were linked to RNA-related processes (Fig. 5a).

Pathway analysis showed that some upregulated proteins were associated with only one term, that is, aminoacyl-tRNA biosynthesis, whereas downregulated proteins were annotated in a total of 16 biological pathways. Amongst these, terms related to oxidative phosphorylation and pathways of neurodegeneration were particularly enriched (Fig. 5b). Results of the overrepresentation analyses are listed in Supplemental Table 3.

### Regional differences in proteome pattern of the white matter in VWM

**Fig. 6:**
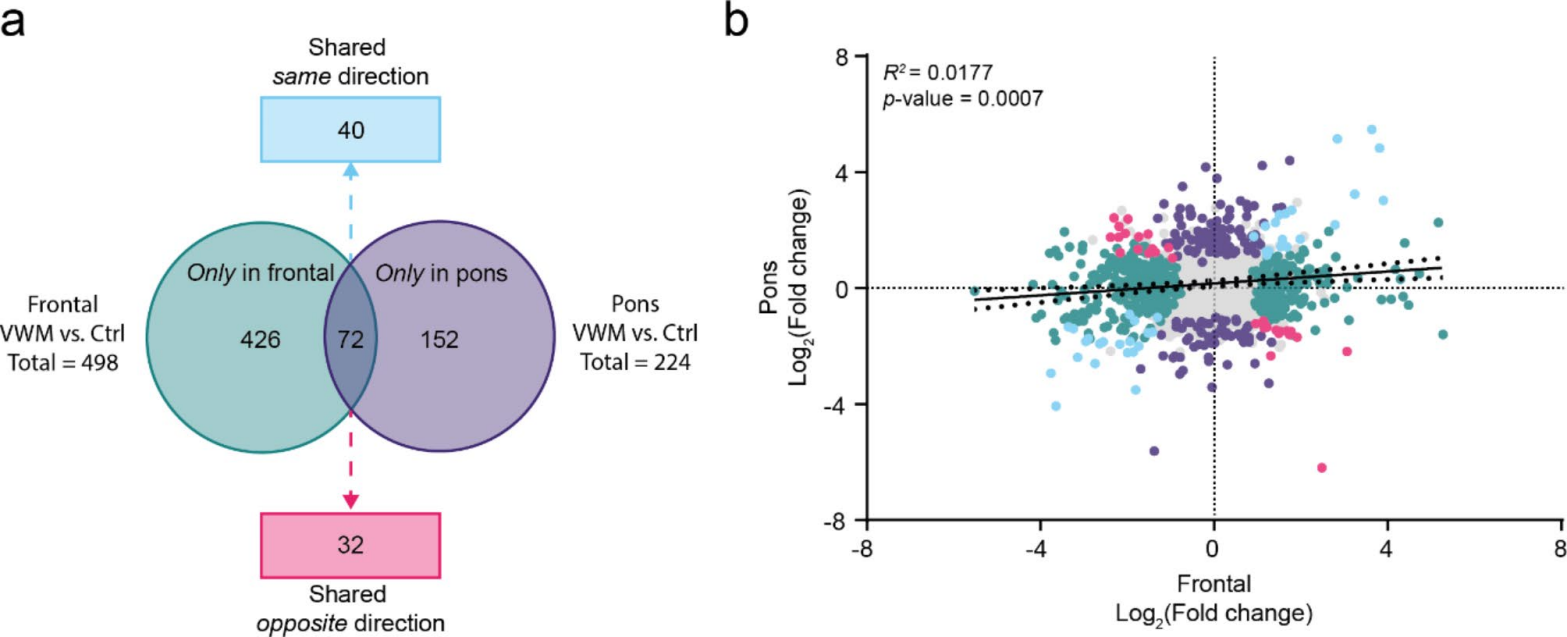
Identifying the overlap of significantly differentially expressed proteins in the VWM frontal and pons white matter. (**a**) Significantly differentially expressed proteins in the VWM frontal (n = 498 proteins) and pons (n = 224 proteins) white matter were compared. Seventy-two proteins were shared between regions. Of these, expression of 40 proteins changed in the same direction (blue), whereas 32 changed in the opposite direction (pink). A total of 426 (green) and 152 (purple) proteins were altered in only the VWM frontal and pons white matter, respectively. (**b**) Correlation analysis of all significantly differentially expressed proteins in the VWM frontal and pons white matter reveals little similarities (*R*^2^ = 0.0177). Significantly altered proteins are highlighted according to the colors in (**a**), and proteins not significantly altered are shown in gray

In VWM, the white matter is predominantly affected. Yet, some brain areas are more vulnerable than others. Comparing the proteome pattern of differently affected brain regions might provide insights into molecular bases underlying this regional vulnerability. After identifying regional differentially expressed proteins in VWM compared to controls (Supplemental Table 2), we performed a side-by-side comparison of the VWM proteome in these 2 white matter regions to determine the overlap of significantly differentially expressed proteins (Fig. 6a and Supplemental Table 4). There was little correlation in protein expression levels (Pearson’s correlation, *R*^2^ = 0.0177, *p*-value = 0.0007), suggesting strong difference in proteome patterns of the white matter in the frontal lobe compared to the pons (Fig. 6b). Indeed, only 40 out of 72 shared differentially expressed proteins were altered in the same direction, suggesting disease mechanisms shared between both regions (Fig. 6a and Supplemental Table 4). Functional overrepresentation analysis of these proteins revealed that those with low expression were enriched for terms associated with brain development, whereas no specific terms were linked to proteins with higher expression (Supplemental Table 5). No biological pathways were found enriched (Supplemental Table 5). We also examined whether these protein changes were associated with particular cell types. Amongst the lower expressed proteins, there was no enrichment for a specific cell type, whereas some higher expressed proteins were enriched in vascular cells (*q*-value = 0.0175) (Supplemental Fig. 3).

**Fig. 7:**
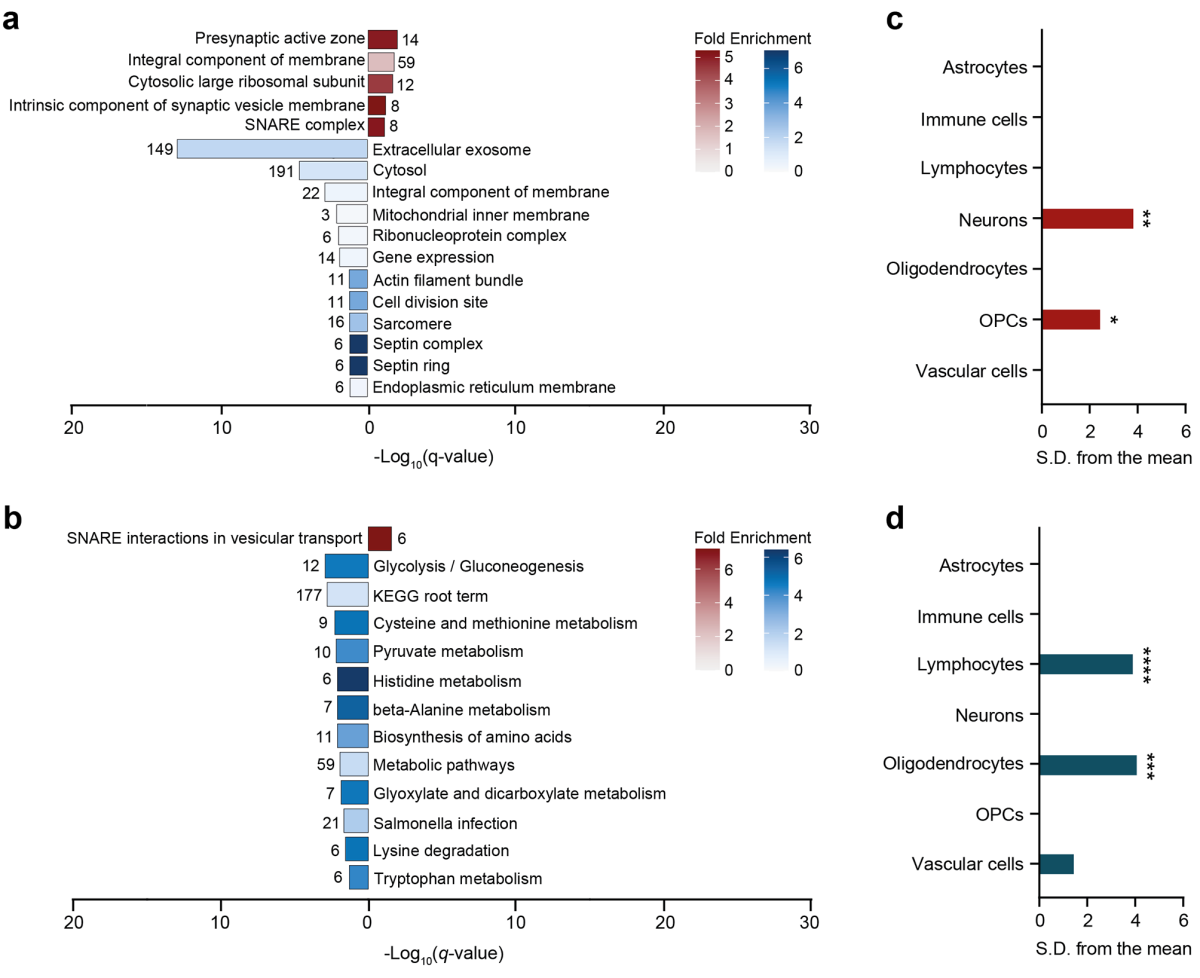
Functional and pathway analysis of proteins distinct for VWM frontal white matter. (**a**) Gene ontology and (**b**) KEGG pathway analysis of proteins downregulated (n = 267) and upregulated (n = 191) in only the VWM frontal white matter. In (**a**), only the most specific ontology subclasses are shown. In (**a-b**), red and blue bars represent terms associated with higher and lower expressed proteins, respectively. Number of proteins annotated in each term is listed in the plot. Fold enrichment values below 1 represent underrepresented terms, whereas a value above 1 represents overrepresented terms. EWCE analysis of the (**c**) upregulated and (**d**) downregulated proteins confirms involvement of distinct cell types in the frontal white matter of VWM patients. * *q*-value < 0.05, ** *q*-value < 0.01, *** *q*-value < 0.001, **** *q*-value < 0.0001, *S.D.* standard deviation, *OPCs* oligodendrocyte progenitor cells

The remaining deregulated proteins represented region-specific changes. Amongst these, 426 and 152 proteins were specifically changed in the frontal and pons white matter in VWM, respectively (Fig. 6a and Supplemental Table 4). Furthermore, 32 proteins showed changes in the opposite direction, that is, upregulation in one region, but downregulation in the other or vice versa (Fig. 6a and Supplemental Table 4). Together, these findings indicate 458 and 174 differentially expressed proteins distinct for the frontal and pons white matter, respectively (Supplemental Table 4). Alterations in these proteins might underlie regional vulnerability in VWM. Functional gene ontology analysis of proteins with increased expression in only the VWM frontal white matter revealed enrichment for terms related to synaptic vesicles (Fig. 7a). Analysis of proteins with decreased expression were enriched for terms associated with cell structure. Notably, terms related to gene expression, membrane, and ribonucleoprotein complex were underrepresented (Fig. 7a). Further pathway analysis suggested that various downregulated proteins were highly associated with glycolysis/gluconeogenesis and amino acid metabolism, whereas upregulated proteins were enriched in SNARE interactions in vesicular transport (Fig. 7b). Cell type enrichment analysis revealed that proteins with increased expression in only VWM frontal white matter were enriched for neurons (*q*-value = 0.0014) and OPCs (*q*-value = 0.0357) (Fig. 7c), whereas proteins with decreased expression were enriched for lymphocytes (*q*-value < 0.0001) and oligodendrocytes (*q*-value = 0.00035) (Fig. 7d). This confirms selective involvement of these cell types in the frontal white matter in VWM.

**Fig. 8:**
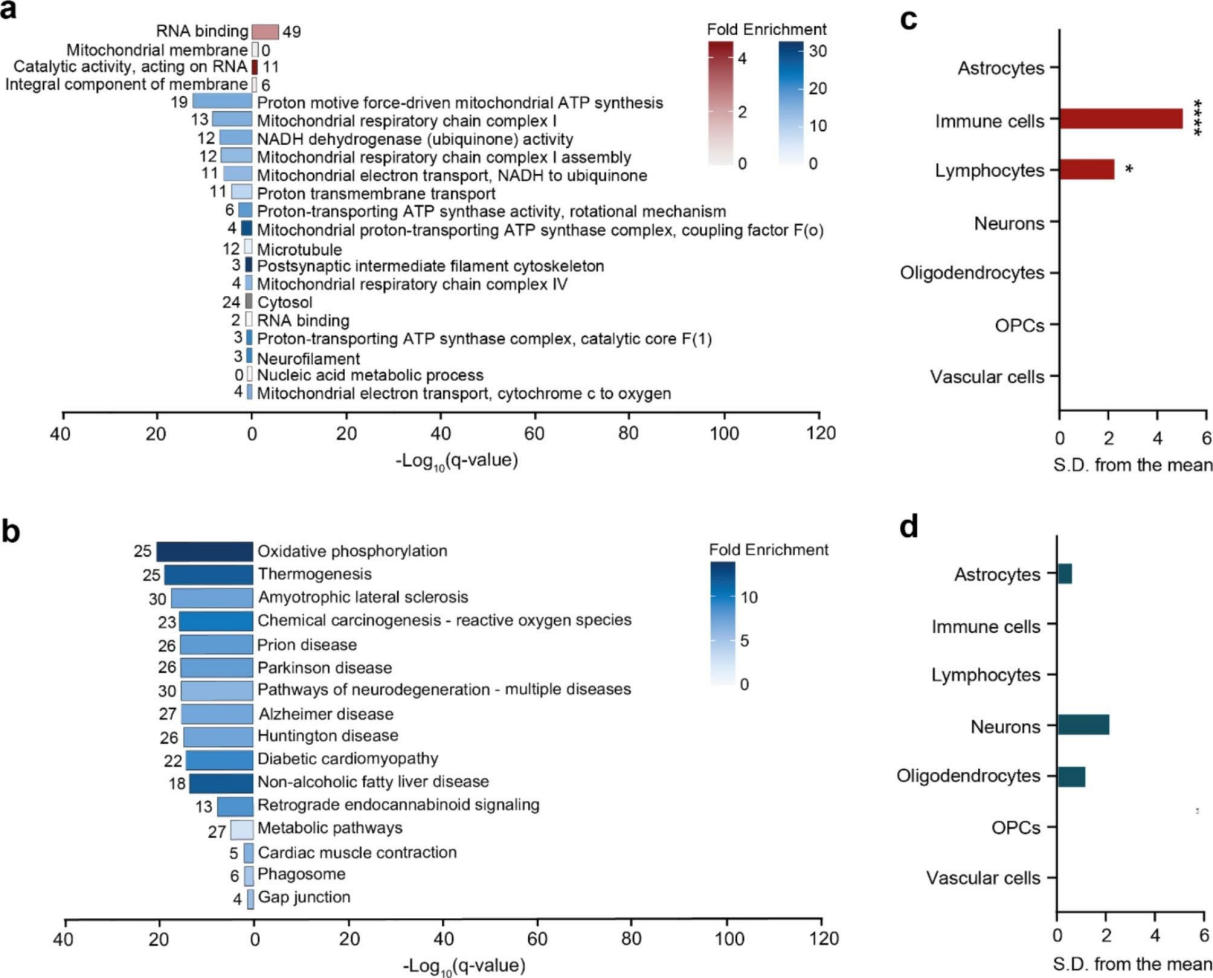
Functional and pathway analysis proteins distinct for VWM pons white matter. (**a**) Gene ontology and (**b**) KEGG pathway analysis of proteins downregulated (n = 72) and upregulated (n = 111) in only the VWM pons white matter. In (**a**), only the most specific ontology subclasses are shown. In (**a-b**), red and blue bars represent terms associated with higher and lower expressed proteins, respectively. Number of proteins annotated in each term is listed in the plot. Fold enrichment values below 1 represent underrepresented terms, whereas a value above 1 represents overrepresented terms. EWCE analysis of the (**c**) upregulated and (**d**) downregulated proteins shows that only proteins with higher expression are associated with specific cell types in the pons white matter of VWM patients. * *q*-value < 0.05, **** *q*-value < 0.0001, *S.D.* standard deviation, *OPCs* oligodendrocyte progenitor cells

Similar analyses were performed with proteins distinct for the pons white matter in VWM. Proteins with decreased expression were enriched in terms, of which the majority were associated to mitochondrial functioning, specifically electron transport-related processes and respiratory chain complexes (Fig. 8a). Pathway analysis revealed that various downregulated proteins were highly enriched for those involved in oxidative phosphorylation and pathways of neurodegeneration (Fig. 8b). Analysis of the proteins with increased expression were enriched for RNA-related terms (Fig. 8a). No biological pathways were associated with proteins that had enhanced expression in only the pons white matter of VWM patients. Results of the overrepresentation analyses are listed in Supplemental Table 5.

Further cell type enrichment analysis revealed that proteins with enhanced expression in only the VWM pons white matter were enriched for immune cells (*q*-value < 0.0001) and lymphocytes (*q*-value = 0.04375), demonstrating involvement of immune cells in this specific region (Fig. 8c). No particular cell types were significantly associated with downregulated proteins (Fig. 8d).

## Discussion

Proteins are responsible for a majority of cellular functions in both health and disease. Mass spectrometry-based proteomics approaches allow unbiased identification of proteins in complex biological samples ranging from whole tissues to even cellular organelles [3]. These approaches are widely used to understand how changes in protein expression, structure and function drive diseases [3]. Relatively little is known about the proteomic landscape of the human VWM brain. In VWM, brain white matter regions vary in susceptibility to pathology [9]. Understanding why some regions are more vulnerable to VWM pathology than others is crucial to elucidate the pathogenesis of the disease. Here, we describe the proteome of the white matter in the frontal lobe and pons in VWM. Our analyses reveal regional patterns of protein changes and also identify affected cell types. These regional patterns are further linked to distinct biological pathways, indicating region-specific pathological features in VWM. Our results show that distinct patterns of protein expression may at least partly explain regional vulnerability to pathology in VWM.

### VWM differently affects brain white matter regions

VWM affects brain regions in a typical pattern with telencephalic areas being most severely affected, whereas other areas remain allegedly spared [9, 37]. In our VWM cohort, all patients presented with comparable severity in white matter pathology in the frontal lobe, and normal imaging and neuropathology of the pons (Supplemental Fig. 1). Indeed, we found 498 differentially expressed proteins in the frontal white matter in VWM patients, confirming substantial changes in comparison to control subjects. Remarkably, we also found an altered proteome with a total of 224 dysregulated proteins in the white matter of the pons, despite showing little if any pathology. Our findings therefore indicate that, rather than being spared, brain regions that appear normal in VWM are instead differently affected. Notably, we found alterations in the expression of myelin-related proteins and astrocyte intermediate filament proteins, of which some are already known to be altered in VWM, supporting the validity of our proteome results. Comparing the proteomes of these brain white matter regions revealed only 40 commonly altered proteins, whereas the remaining proteins (~ 94%) showed region-specific alterations, confirming that VWM indeed differently affects brain regions. Consistent with this, we have previously shown that the frontal cortex in VWM, traditionally considered to be spared, is also affected with alterations in protein composition, and comparable, but less severe, neuropathological features as in the brain white matter [23]. Together, our findings show that brain regions that appear normal in VWM are not normal.

### Region-specific involvement of brain cell types in VWM

Given that proteins are responsible for normal cellular functioning, region-specific changes in protein composition could trigger alterations at the cellular level. In line with the known neuropathology of more and less affected VWM white matter regions, our data show that overexpressed proteins in the VWM frontal white matter are enriched for neurons and OPCs, whereas proteins with decreased expression are enriched for lymphocytes and mature oligodendrocytes. In the frontal white matter, there is indeed a remarkably high density of OPCs, absence of mature oligodendrocytes, and lack of immune cells and lymphocytes [9]. Enrichment for neurons could probably reflect preservation of white matter neurons in VWM. By contrast, the protein signature of the VWM pons white matter indicates involvement of immune cells (microglia and macrophages) and lymphocytes, indicative of inflammation. Inflammation is typically not found in more severely affected areas, and could reflect region-specific cellular pathology [9]. Indeed, inflammation is observed in relatively spared areas such as the pons, possibly limiting the degree of injury [34, 38]. Future studies should investigate the role of inflammatory processes in regional vulnerability in VWM.

Notably, in VWM, astrocytes are pointed out as the driving forces of the pathogenesis, and trigger secondary effects in axons and oligodendrocytes also via secreted factors [7, 8, 11, 20]. Previous studies have identified the glycosaminoglycan hyaluronan as a highly secreted factor by astrocytes that inhibits oligodendrocyte maturation in VWM [8, 11]. Interestingly, hyaluronan accumulation differs across brain white matter, with highest accumulation in the most severely affected areas, suggesting that astrocytes may contribute to regional vulnerability to VWM pathology [8]. We, however, did not find any regional protein changes associated with astrocytes in our study. An explanation for this discrepancy may be the use of postmortem tissue. This often reflects the end-stage of the disease, and possibly only shows secondary effects of the primarily affected cell type (i.e., astrocytes) on other cell types [33].

### Region-specific involvement of biological processes in VWM

The majority of the dysregulated proteins are region-specific, that is either altered in the white matter of the frontal lobe, but not in the pons or vice versa. These regional protein changes correspond to several processes, including synaptic transmission, RNA-related processes, and pathways involved in cellular respiratory metabolism. Deficits in cellular respiratory metabolism have been implicated in VWM [12, 15, 16, 23, 30]. Previous studies showed that mitochondrial respiration is impaired in *Eif2b*-mutant mouse fibroblasts, oligodendrocytes, and astrocytes with increase in mitochondrial abundance and glycolysis, and decrease in oxidative respiration components [15, 16, 30]. Our data show alterations in glycolysis/gluconeogenesis in the frontal white matter. These metabolic processes represent the first stages of cellular respiration, in which glucose is degraded (glycolysis) or synthesized (gluconeogenesis) [13, 14, 32]. The decrease in glycolysis/gluconeogenesis in the white matter of the frontal lobe contrasts with previous findings observed in preclinical VWM mouse models [12, 15, 16, 30]. This discrepancy could be due to species-specific differences between humans and rodents. How impairment in the first stage of cellular respiration drives white matter pathology in VWM is unknown. Although it is not clear whether there is an impaired glucose consumption or utilization in VWM, an adequate glucose metabolism is essential for OPC maturation and myelin synthesis and maintenance [24]. We speculate that the decrease in glucose metabolic processes could be a contributor to halted OPC differentiation and maturation in the VWM white matter.

In addition to alterations in glucose metabolism, we found changes in amino acid metabolism in the frontal white matter. Amino acids contribute to cellular respiratory metabolism via the glycolysis and tricarboxylic acid (TCA) cycle [4, 13, 14, 27, 32]. Deficits in amino acid metabolic processes have been implicated in VWM before. For example, a higher level of amino acids serine and glycine have been found in the cerebrospinal fluid of VWM patients [42]. Increased amino acid transport and serine, glycine and cysteine biosynthesis as well as higher levels of asparagine and glycine have also been reported in VWM-mutant mice brain, and are allegedly linked to ISR deregulation [1, 26, 42]. We now add to these findings and show that proteins with decreased expression in the frontal white matter are involved in the metabolism of several classes of amino acids in the VWM frontal white matter. Amongst these were cysteine and methionine, histidine, beta-alanine, lysine, and tryptophan. It remains unclear how these metabolic changes affect the VWM frontal white matter, but amino acid metabolism participates in coordinating the optimal energetic balance needed for all white matter cellular activities [37].

We also found changes in mitochondrial oxidative phosphorylation. During oxidative phosphorylation oxidation/reduction reactions take place, followed by the release of energy in the form of adenosine triphosphate (ATP) [13, 14, 32]. In the VWM pons white matter, we found a decreased expression of proteins encoding components within mitochondrial respiratory chain complexes, suggestive of a reduction in oxidative phosphorylation. This is consistent with previous studies in VWM mouse models [15, 16, 30], supporting defects in oxidative phosphorylation in VWM. Taken together, our data suggest that different stages of cellular respiration are deregulated across affected white matter areas in VWM. These region-specific changes in cellular respiratory pathways could in part contribute to regional susceptibility in VWM brain white matter. Future studies are warranted to further investigate how different stages of cellular respiratory metabolism determine regional disease severity in VWM.

### Limitations

Some limitations apply to this work. A potential concern is the mismatch in ages between VWM patients and controls. Control brain tissue without confounding neuropathology, obtained at short post mortem time from young donors and children, is extremely rare. In particular, the long post mortem time at which the tissue is harvested elsewhere makes it unsuitable for selected experiments, including proteomics. It should, therefore, be noted that given the active myelination present in the brains of younger individuals, it is possible that certain proteome changes detected in this study may in part be caused by aging.

Furthermore, our proteome analysis was focused on regional changes in the abundance of proteins that are relevant to VWM pathogenesis. It would be of interest to further explore other white matter disorders in general and leukodystrophies in particular to gain insight into the possible commonalities and differences between different diseases at the protein level.

## Conclusion

In conclusion, our novel proteomic data shed light on the pathophysiology of VWM in different ways. They show that relatively or allegedly completely spared brain regions are affected in VWM at a molecular level, in line with previous observations concerning the cortex. The data also pinpoint the basic cellular molecular pathways and components that are differentially affected in the VWM brain. Further studies are warranted to explore how this information translates to a cellular functional level.

## Electronic supplementary material

Below is the link to the electronic supplementary material.

Supplementary Material 1: **Supplemental Table 1** List of all unique proteins detected and quantified across the white matter of the frontal lobe and pons in control and VWM cases

Supplementary Material 2: **Supplemental Table 2** List of significantly differentially expressed proteins in the white matter of the frontal lobe and pons in VWM compared to control

Supplementary Material 3: **Supplemental Table 3** Results of gene ontology and pathway analysis of significantly differentially expressed proteins in VWM frontal and pons white matter

Supplementary Material 4: **Supplemental Table 4** Comparison of proteins with differential expression in the frontal and pons white matter in VWM

Supplementary Material 5: **Supplemental Table 5** Results of gene ontology and pathway analysis of all 650 protein found significantly differentially expressed across the frontal and pons white matter in VWM

Supplementary Material 6: **Supplemental Fig. 1** Prototypic MRI of a 32-year-old VWM patient with age of onset at 7 years. (**a-b**) T2-weighted images show (**a**) a normal pons and (**b**) a severe cerebral white matter disease with diffuse signal abnormality and atrophy. (**c**) FLAIR image shows that the cerebral white matter is largely rarefied. Red arrows indicate in (**a**) the normal pons and in (**b-c**) the affected cerebral white matter

Supplementary Material 7: **Supplemental Fig. 2** Principal component analyses of all white matter in the frontal lobe and pons in control and VWM cases. Analysis of all samples reveal separation of VWM frontal white matter samples from all other samples in the first component, explaining 29.5% of the variability. The second component separates control pons white matter samples from control frontal as well as pons white matter samples. This accounts for 17.0% of the variability. *PC1* principal component 1, *PC2* principal component 2, *Ctrl* control, *VWM* vanishing white matter

Supplementary Material 8: **Supplemental Fig. 3** EWCE analysis of proteins found altered in the same direction in both the frontal and pons white matter in VWM (n = 40). Analysis of (**a**) upregulated and (**b**) downregulated proteins. * *q*-value < 0.05

## Data Availability

Proteomics data have been deposited into the ProteomeXchange Consortium via the PRIDE partner repository with the dataset identifier PXD040861 [10, 28].
