# Supplementary figures and images for: Regional vulnerability of brain white matter in vanishing white matter
Source: Acta Neuropathol Commun. 2023 Jun 22;11:103. doi: 10.1186/s40478-023-01599-6 (PMC10286497; doi:10.1186/s40478-023-01599-6)

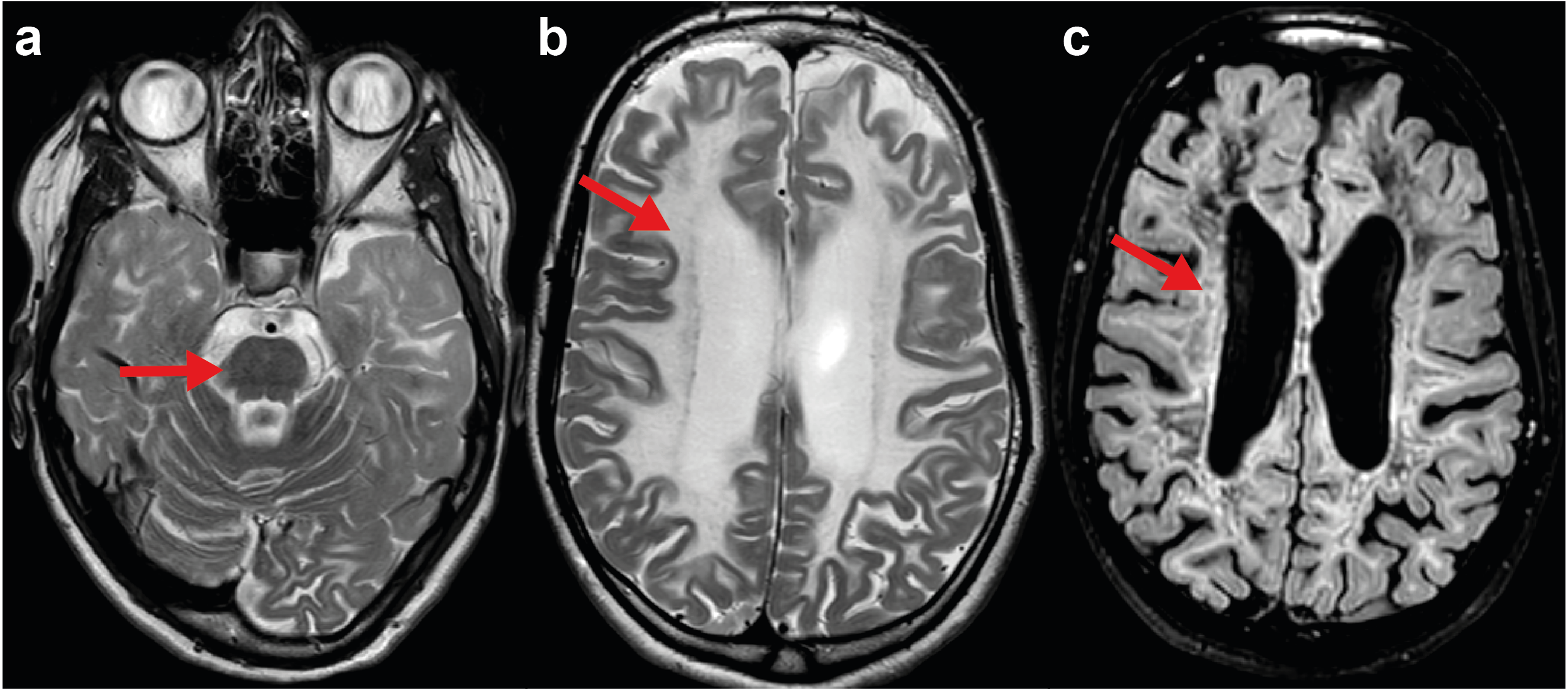

Supplement: Supplementary file 6 — Supplementary Material 6: Supplemental Fig. 1 Prototypic MRI of a 32-year-old VWM patient with age of onset at 7 years. (a-b) T2-weighted images show (a) a normal pons and (b) a severe cerebral white matter disease with diffuse signal abnormality and atrophy. (c) FLAIR image shows that the cerebral white matter is largely rarefied. Red arrows indicate in (a) the normal pons and in (b-c) the affected cerebral white matter [file 40478_2023_1599_MOESM6_ESM.png]

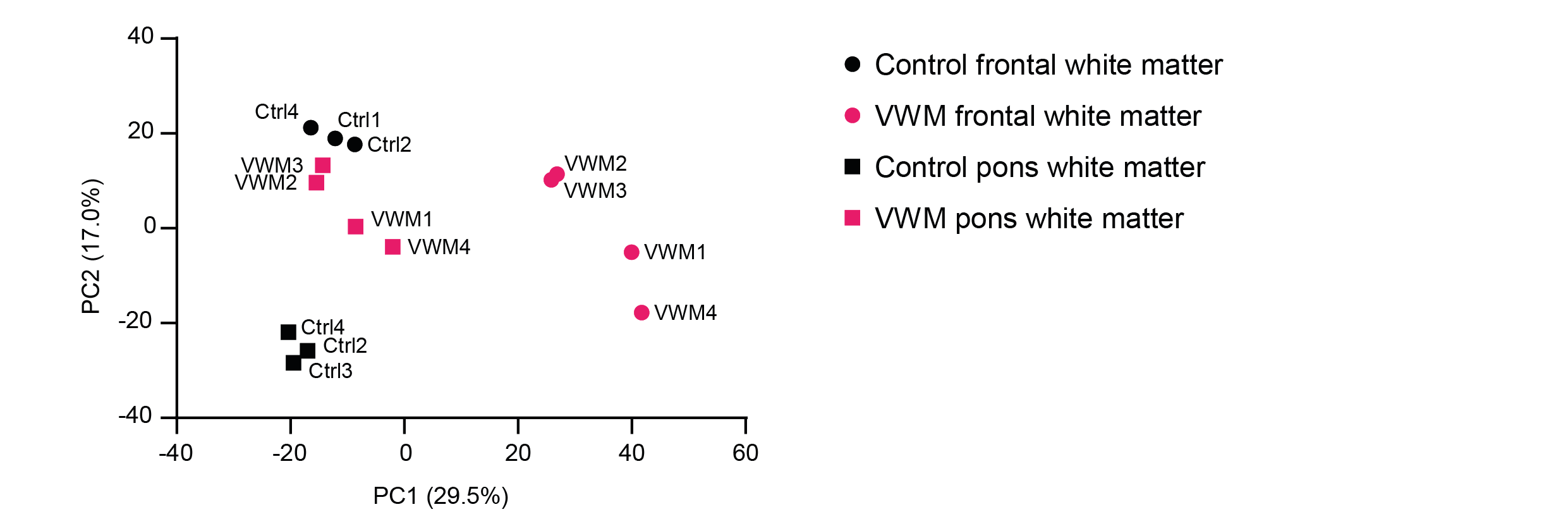

Supplement: Supplementary file 7 — Supplementary Material 7: Supplemental Fig. 2 Principal component analyses of all white matter in the frontal lobe and pons in control and VWM cases. Analysis of all samples reveal separation of VWM frontal white matter samples from all other samples in the first component, explaining 29.5% of the variability. The second component separates control pons white matter samples from control frontal as well as pons white matter samples. This accounts for 17.0% of the variability. PC1 principal component 1, PC2 principal component 2, Ctrl control, VWM vanishing white matter [file 40478_2023_1599_MOESM7_ESM.png]

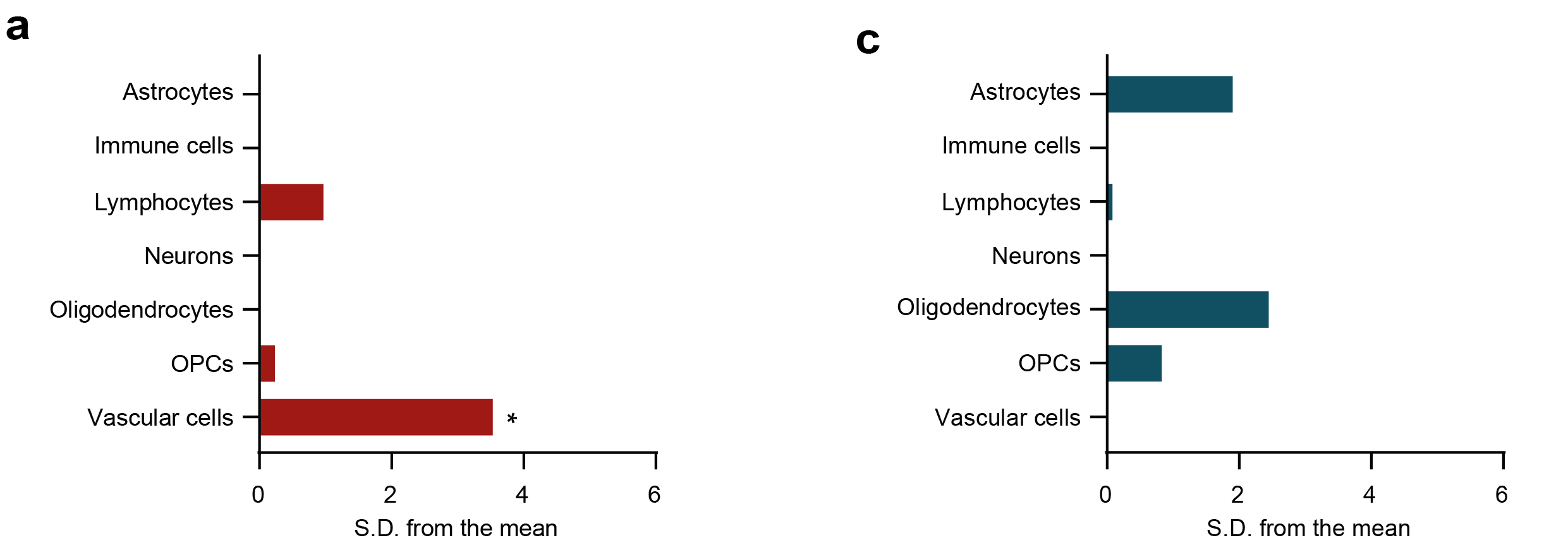

Supplement: Supplementary file 8 — Supplementary Material 8: Supplemental Fig. 3 EWCE analysis of proteins found altered in the same direction in both the frontal and pons white matter in VWM (n = 40). Analysis of (a) upregulated and (b) downregulated proteins. * q-value < 0.05 [file 40478_2023_1599_MOESM8_ESM.png]
